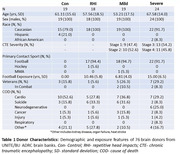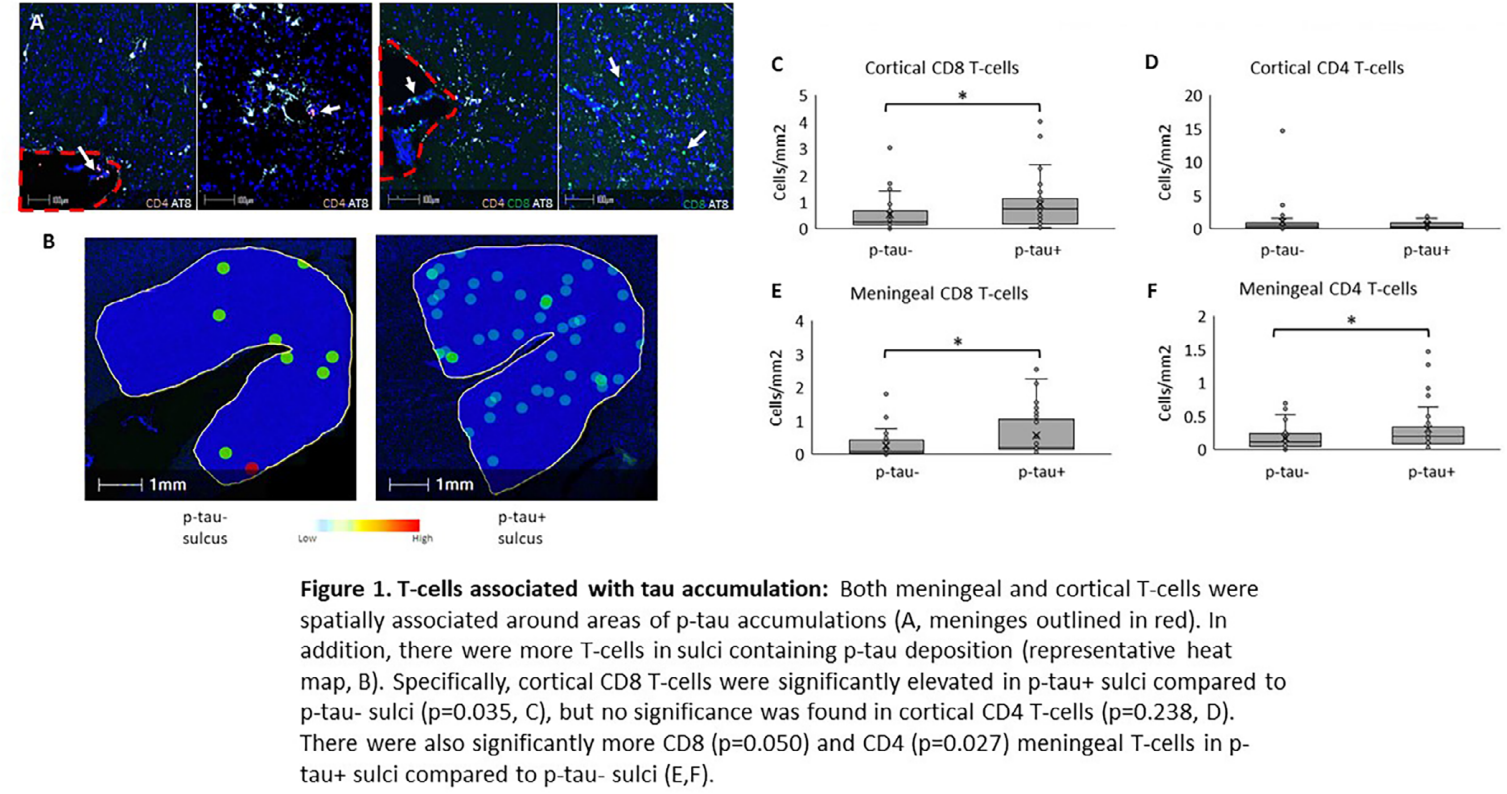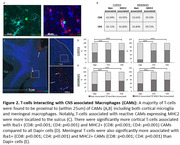# Meningeal and infiltrating T‐cells are associated with neuroinflammation after head injury and with tau pathology in CTE

**DOI:** 10.1002/alz.089160

**Published:** 2025-01-03

**Authors:** Samantha M Calderazzo, Thor D. Stein, David M. Holtzman, Ann C. McKee, Bertrand Russell Huber

**Affiliations:** ^1^ Dept of Pathology and Laboratory Medicine, Boston University School of Medicine, Boston, MA USA; ^2^ Chronic Traumatic Encephalopathy Center Boston University, Boston, MA USA; ^3^ Boston University Alzheimer’s Disease Research Center, Boston, MA USA; ^4^ VA Boston Healthcare System, Boston, MA USA; ^5^ Department of Pathology and Laboratory Medicine, Boston University Chobanian & Avedisian School of Medicine, Boston, MA USA; ^6^ Washington University School of Medicine in St Louis, St. Louis, MO USA; ^7^ Hope Center for Neurological Disorders, Washington University School of Medicine, St. Louis, MO USA; ^8^ VA Bedford Healthcare System, Bedford, MA USA; ^9^ Boston University Chronic Traumatic Encephalopathy Center, Boston University Chobanian & Avedisian School of Medicine, Boston, MA USA; ^10^ Department of Neurology, Boston University Chobanian & Avedisian School of Medicine, Boston, MA USA

## Abstract

**Background:**

T‐cell infiltration into the brain parenchyma is associated with hyperphosphorylated tau (p‐tau) accumulation in neurodegenerative diseases. Chronic traumatic encephalopathy (CTE) is a progressive tauopathy caused by exposure to repetitive head impacts (RHI). CTE is defined by the perivascular accumulation of p‐tau at the cortical sulcal depths and can be stratified into mild and severe pathological stages. RHI exposure and CTE are associated with progressive microglial inflammation, but the involvement of T cells is unknown.

**Method:**

The dorsolateral frontal cortex from 76 brain donors in the UNITE/ BU ADRC brain bank was analyzed, all men (age 30‐80 yrs), including 19 with mild CTE (stage 1‐2), 24 with severe CTE (stage 3‐4),18 with RHI exposure without CTE, and 19 without RHI exposure or neurodegenerative disease (Table 1). Years of football served as a proxy for the duration of RHI. Multiplex immunofluorescence for T‐cells (CD8 1:20, CD4 1:100), endothelial cells (CD31 1:200), microglia (iba1 1:500, MHC2 1:500), and p‐tau (AT8 1:500) were used. Synaptic markers (PSD95 1:100, Gephyrin 1:200, VGlut 1:1200, VGat 1:1500) were used on adjacent tissue sections to assess synaptic loss. Four regions were annotated: leptomeninges, sulcus (gray matter (gm) in bottom third of gyrus), crest (gm in top third of gyrus), and white matter. Slides were digitally scanned, and HALO software was used to quantify T‐cell numbers and spatial relationships. ANCOVA with posthoc Bonferroni corrections were performed for between group analyses; multivariate linear correlations were utilized for associations between T‐cells and other markers or RHI exposure, all using age as a covariate.

**Result:**

Infiltrating T‐cells were significantly increased at the sulcus in RHI, mild, and severe CTE, with distinct subtypes predominating in RHI and CTE. In addition, T‐cells were correlated with the duration of RHI exposure and synaptic loss. Meningeal and infiltrating T‐cells were notably elevated in p‐tau positive sulci (Fig. 1). Finally, T‐cells were spatially related to CNS‐associated macrophages expressing MHC2, including cortical microglia and meningeal macrophages (Fig. 2).

**Conclusion:**

These data suggest an adaptive immune T‐cell response occurs with RHI exposure, which might be microglia mediated and contribute to tau neurodegeneration in CTE.